# Glucose metabolism and NRF2 coordinate the antioxidant response in melanoma resistant to MAPK inhibitors

**DOI:** 10.1038/s41419-018-0340-4

**Published:** 2018-02-27

**Authors:** Raeeka Khamari, Anne Trinh, Pierre Elliott Gabert, Paola Corazao-Rozas, Samuel Riveros-Cruz, Stephane Balayssac, Myriam Malet-Martino, Salim Dekiouk, Marie Joncquel Chevalier Curt, Patrice Maboudou, Guillaume Garçon, Laura Ravasi, Pierre Guerreschi, Laurent Mortier, Bruno Quesnel, Philippe Marchetti, Jerome Kluza

**Affiliations:** 1 Univ. Lille, INSERM, UMR–S1172, Jean Pierre Aubert Research Centre, 59045 Lille, France; 2 Laboratoire SPCMIB UMR CNRS 5068, Université Paul Sabatier, 118 Route de Narbonne, 31062 Toulouse Cedex 9, France; 3 Department of Biochemistry and Molecular Biology, CHU Lille, Lille, France; 4 Centre de Biologie-Pathologie-Génétique, CHU Lille, Lille, France; 5 EA4483-IMPECS, Institut Pasteur de Lille, Univ. Lille, CHU Lille, Lille, France; 6 In Vivo Preclinical Imaging Facility, IFR 114 - University of Lille, Nord of France, CHU Lille, Lille, France; 7 Université Lille, INSERM Unité 1189, CHU Lille, Lille, France; 8 Service des Maladies du Sang, CHU Lille, F-59000 Lille, France; 9 Centre de Biologie-Pathologie-Génétique Banque de Tissus et UF8793, CHU Lille, Lille, France

## Abstract

Targeted therapies as BRAF and MEK inhibitor combination have been approved as first-line treatment for BRAF-mutant melanoma. However, disease progression occurs in most of the patients within few months of therapy. Metabolic adaptations have been described in the context of acquired resistance to BRAF inhibitors (BRAFi). BRAFi-resistant melanomas are characterized by an increase of mitochondrial oxidative phosphorylation and are more prone to cell death induced by mitochondrial-targeting drugs. BRAFi-resistant melanomas also exhibit an enhancement of oxidative stress due to mitochondrial oxygen consumption increase. To understand the mechanisms responsible for survival of BRAFi-resistant melanoma cells in the context of oxidative stress, we have established a preclinical murine model that accurately recapitulates in vivo the acquisition of resistance to MAPK inhibitors including several BRAF or MEK inhibitors alone and in combination. Using mice model and melanoma cell lines generated from mice tumors, we have confirmed that the acquisition of resistance is associated with an increase in mitochondrial oxidative phosphorylation as well as the importance of glutamine metabolism. Moreover, we have demonstrated that BRAFi-resistant melanoma can adapt mitochondrial metabolism to support glucose-derived glutamate synthesis leading to increase in glutathione content. Besides, BRAFi-resistant melanoma exhibits a strong activation of NRF-2 pathway leading to increase in the pentose phosphate pathway, which is involved in the regeneration of reduced glutathione, and to increase in xCT expression, a component of the xc—amino acid transporter essential for the uptake of cystine required for intracellular glutathione synthesis. All these metabolic modifications sustain glutathione level and contribute to the intracellular redox balance to allow survival of BRAFi-resistant melanoma cells.

## Introduction

Activating V600E/K mutations in the BRAF oncogene are found in over half of the patients with metastatic melanoma. These mutations confer constitutive activation of BRAF kinase and drive oncogenic signaling through MAPK activation. Targeted therapies as BRAF and MEK inhibitors combination (e.g., the BRAF inhibitors vemurafenib and the MEK inhibitor trametinib) have revolutionized the treatment of patients leading to increase progression-free survival and overall survival. Unfortunately, disease progression occurs after a median of few months and in 80% of patients after 3 years of therapies^1^. Immune checkpoint inhibitors (as PD-L1/PD-1 or CTLA-4 monoclonoal antibodies) have also shown activity in some patients with BRAFV600E-mutant melanoma, but most of the patients progressed on this treatment. Clinical trials are actually evaluating combination of both MAPKinase inhibitors and PD-1/PD-L1 antagonists^2^.

In the context of MAPK constitutive activation, mutant BRAF stimulates glycolytic activity and inhibits mitochondrial oxidative phosphorylation^3^. BRAF inhibition causes a decrease in ERK activation resulting in G1 phase cell cycle arrest and inducing endoplasmic reticulum (ER)-stress-mediated cell death^4–6^. We and others have shown that MAPK inhibitors reverse also the metabolic phenotype by decreasing glycolytic activity and increasing mitochondrial oxidative phosphorylation (OXPHOS) of BRAF-driven melanoma cells^5,7,8^. These modifications of mitochondrial metabolism following MAPK inhibition could be considered as an adaptive response to compensate drug-induced glycolytic inhibition. In agreement with this observation, drugs repressing directly or indirectly mitochondrial oxidative metabolism favorized cell death under BRAFi therapy^5,9–13^.

Several mechanisms of acquired resistance to BRAFi have been identified, mostly mediated by MAPK pathway reactivation including mutations in NRAS and MEK oncogenes, BRAFV600E splice variants, and BRAF amplification or activation of alternative tumorigenic pathway. We and others have also shown that vemurafenib-resistant melanoma cells maintain an increase of mitochondrial oxidative phosphorylation even in absence of BRAFi^9,10^. Marais and coll have shown BRAFi that resistant melanoma cells support mitochondrial respiration and ATP supply by sustaining TCA cycle metabolites levels using glutaminolysis^10^. Oxygen consumption increase leads to mitochondrial ROS overproduction through electron transfer chain and to enhancement of oxidative stress. As a weakness in spite of overall strength, we have demonstrated that BRAFi-resistant cells with increased endogenous ROS are more sensitive to cell death upon exposure to mitochondrial pro-oxidative agents^9^.

Here, we asked the question how BRAFi-resistant melanoma cells use metabolism to cope with ROS production and therefore to survive under oxidative stress. To understand the mechanisms leading to BRAFi-resistant melanoma cells survival in this context, we have generated mouse models bearing human BRAFV600E melanoma cells that mimic clinical relapse and acquired resistance to BRAF inhibitors.

## Results

**Fig. 1:**
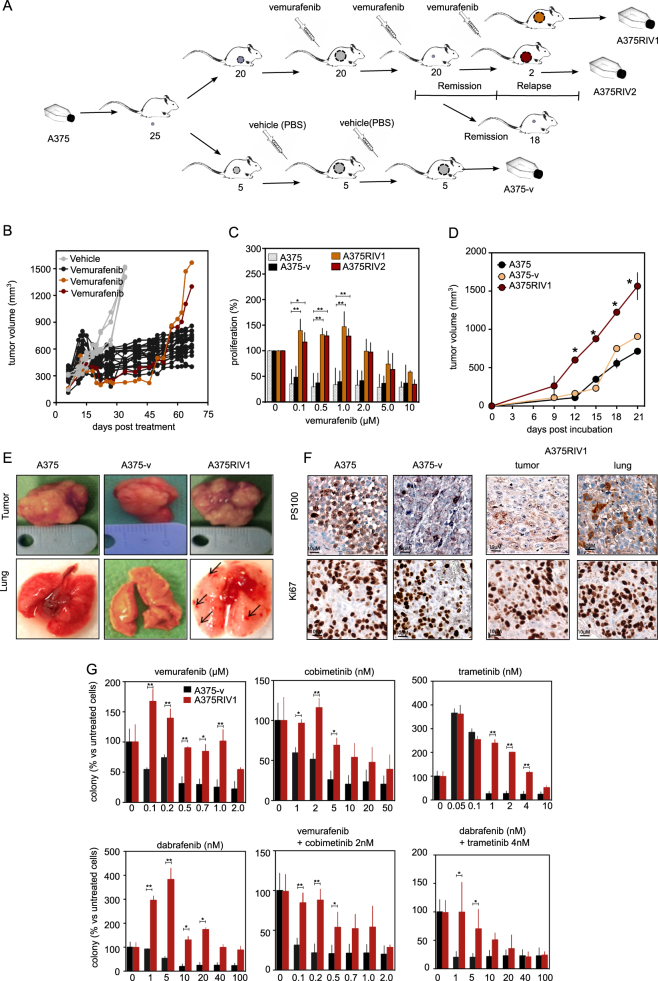
Conception and characterization of MAPK-resistant BRAFV600E melanoma model from SCID mice. **a** Summary of the experimental procedure used to generate melanoma cell lines used in this study. Briefly, 25 SCID mice have been inoculated with A375 melanoma cells. Five mice were treated with vehicle only and killed when tumors have reached 1500 mm^3^. A375-v cells have been obtained after tumor dissociation from one of these tumors. Twenty other mice were treated with vemurafenib leading to a rapid tumor shrinkage. Forty days after the beginning of the treatment, two mice have exhibited tumor progression under vemurafenib therapy. Tumors have been extracted and have been dissociated to generate A375RIV1 and A375RIV2 cell lines. **b** Tumor progression of A375-injected mice treated with vehicule only (*n* = 5) or with vemurafenib as indicated in materials and method. **c** Proliferation of A375, A375-v, A375RIV1, and A375RIV2 cell lines exposed in vitro to vemurafenib at the indicated concentration for 72 h. The values represent the mean ± SD of three independent experiments. Statistical analyses were performed by two-way ANOVA with a 95% interval of confidence followed by Bonferroni’s post-test. *P < 0.05 and **P < 0.01. **d** Tumor progression of A375, A375-v, and A375RIV1-injected mice (mean ± SD; *n* = 5, statistical analyses were performed compared to A375 by two-way ANOVA with a 95% interval of confidence followed by Bonferroni’s post-test. *P < 0.05 and **P < 0.01). **e** Representative macroscopic views illustrating lungs and primary tumors in A375, A375-v, and A375RIV1-injected mice. Arrows indicate metastasis. **f** Sections from the primary tumors of A375, A375-v, and A375RIV1-injected mice or from lung metastasis of A375RIV1-injected mice. Samples were stained with PS100 to confirm immuno-histological profile of melanoma or with Ki67 antibody to assess proliferation. **g** Colony-forming ability of A375-v and A375RIV1 treated with indicated doses of vemurafenib, cobimetinib, trametinib, dabrafenib, or combination of vemurafenib/cobimetinib or debrafenib/trametinib for 7 days. The values represent the mean ± SD of three independent experiments. Statistical analyses were performed by two-way ANOVA with a 95% interval of confidence followed by Bonferroni’s post-test. *P < 0.05 and **P < 0.01

To generate in vivo melanoma model resistant to BRAFi, SCID mice were engrafted with A375 melanoma cells (Fig. 1a). When tumors reached 150 mm^3^, mice were divided into two groups. One group (*n* = 20) was treated with vemurafenib (300 mg kg^−1^, bid) and the other one (*n* = 5) was treated with vehicle only (PBS). In vemurafenib-treated mice, tumors have drastically reduced until to be undetectable at 30 days post treatment (Fig. 1b). At the opposite, tumors have exponentially increased in vehicle-treated mice. As observed in patients who relapsed after treatment, cancer cells are finally able to escape to anti-BRAF therapy and tumors have re-initiated growth in two mice. Then, mice have been killed and cancer cells have been isolated from tumors to generate three cell lines: A375-v and A375RIV1/A375RIV2 from vehicle-treated mice and vemurafenib-treated mice, respectively. As expected, clonogenic assays confirm that A375RIV1 and A375RIV2 cell lines were more resistant to vemurafenib than A375 or A375-v (Fig. 1c). To confirm the behavior of these cells in vivo, cell lines were engrafted in SCID mice and tumor volumes were periodically measured. As seen in Fig. 1d, A375 and A375-v tumors exhibited the same growth rate while A375RIV1 volume increased faster. Moreover, unlike A375 or A375-v engrafted mice, macroscopic and histological lung analysis showed the presence of many metastases in A375RIV1-engrafted mice indicating a more aggressive phenotype (Fig. 1e, f). Finally, A375RIV1 cell lines were also resistant to other BRAFi (dabrafenib) and MEK inhibitors (cobimetinib and trametinib) alone or in combination (Fig. 1g). Thus, we generated in vivo models for relapsed melanoma cells, which mimic the clinical process of melanoma in humans treated with BRAFi.

**Fig. 2:**
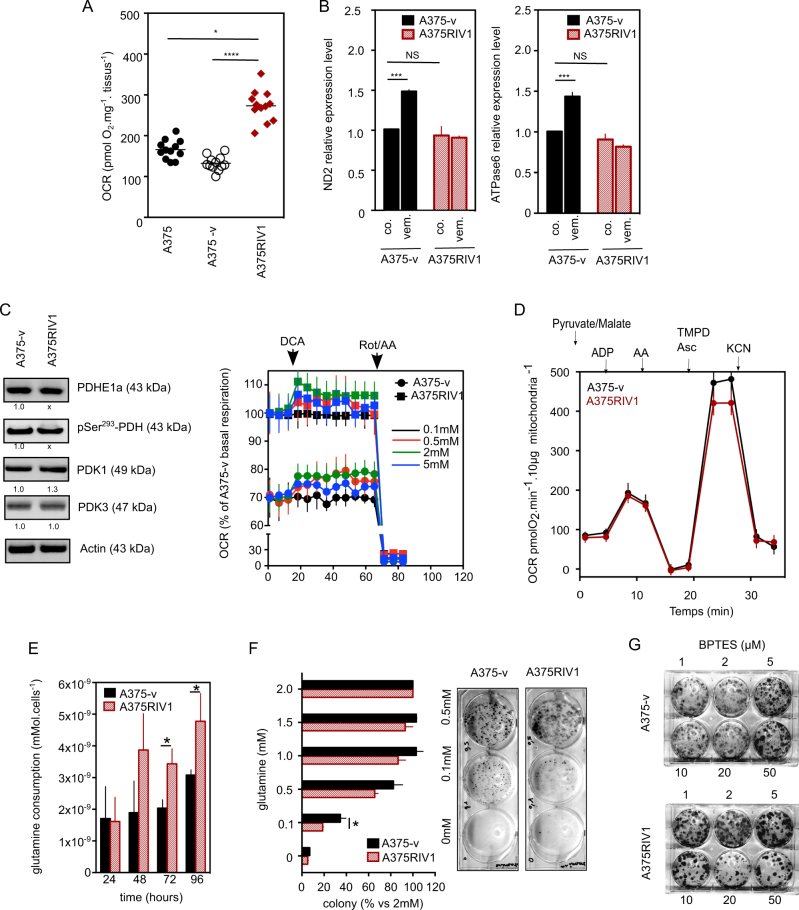
MAPK-resistant melanoma exhibits OXPHOS dependency and glutamine addiction. **a** Oxygen consumption rate (OCR pmol O_2_/min/mg tissues) from tumor biopsy obtained in A375, A375-v, and A375RIV1-injected mice (mean ± SD; *n* = 12, statistical analysis was performed by one-way ANOVA with a 95% interval of confidence followed by Bonferroni’s post-test. *P < 0.05 and ***P < 0.005). **b** Analysis of relative mitochondrial DNA copy number in A375-v or A375RIV1 tumor biopsy by assessment of mtDNA-encoded ND2 RNA (left panel) or ATPase6 RNA. The transcripts level in each sample was normalized to that of ATPSynthB RNA. Mean ± SD; *n* = 3, statistical analyses were performed by two-way ANOVA with a 95% interval of confidence followed by Bonferroni’s post-test. ***P < 0.005). **c** Left panel: oxygen consumption rate (OCR pmol/min/20 000 cells) in A375-v or A375RIV1 cells. PDK inhibitor DCA (0.1, 0.5, 2, or 5 mM) has been injected when indicated (black arrow); right panel: immunoblotting of pyruvate dehydrogenase E1 component subunit alpha (PDHE1a), phospho-serine 293 of PDHE1a, pyruvate dehydrogenase kinase 1 (PDK1), and pyruvate dehydrogenase kinase 3 (PDK3) in A375-v cells compared to A375RIV1. Actin served as loading control. **d** Oxygen consumption rate (OCR pmol/min/10 µg mitochondria) of isolated mitochondria obtained from tumor of A375-v and A375RIV1-injected mice. Mitochondria have been incubated in a pyruvate/malate buffer and the following molecules have been injected when indicated: adenosine 5′-diphosphate sodium (ADP), antimycin A (AA), tetramethyl-p-phenylenediamine/ascorbate (TMPD/Asc), and potassium cyanide (KCN) (*n* = 3). **e** Glutamine consumption of A375-v or A375RIV1 cell lines growing for 24, 48, 72, or 96 h in full medium (mean ± SD; *n* = 3, statistical analyses were performed by two-way ANOVA with a 95% interval of confidence followed by Bonferroni’s post-test. *P < 0.05). **f** Colony-forming ability of A375-v and A375RIV1 growing in medium containing the indicated glutamine concentration for 7 days (mean ± SD; *n* = 3, statistical analyses were performed by two-way ANOVA with a 95% interval of confidence followed by Bonferroni’s post-test. *P < 0.05). **g** Colony-forming ability of A375-v and A375RIV1 exposed to glutaminase (GLS) inhibitor BPTES in full medium for 7 days. Pictures are representative of three independent experiments

**Fig. 3:**
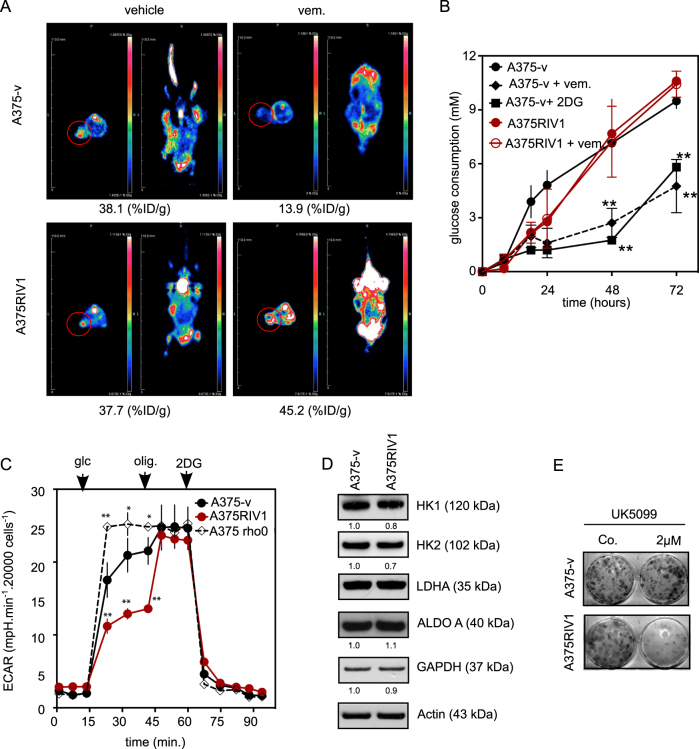
Mitochondrial pyruvate uptake increases in MAPK-resistant melanoma cells. **a** Coronal and sagittal sections of A375-v or A375RIV1-injected mice xenograft scan 1 h after single 18F-FDG injection using microPET to show change in tumor activity and tracer distribution. As indicated, mice have been treated with vehicle or with vemurafenib for 7 days. Mean %ID/g values are shown below to tumor xenograft. **b** Glucose consumption of A375-v or A375RIV1 cell lines growing for 24, 48, or 72 h in full medium in the absence (Co.) or presence of 0.3 µM of vemurafenib. 2-deoxyglucose (2DG) has been used as positive control to inhibit glucose uptake in A375-v cells (mean ± SD; *n* = 3, statistical analyses were performed by two-way ANOVA compared to A375-v with a 95% interval of confidence followed by Bonferroni’s post-test. **P < 0.001). **c** Glycosis activity in A375-v, A375RIV1, and A375rho0 has been assessed by measuring ECAR (mpH min^−1^ 20,000 cells) with Seahorse technology. All cell lines have been incubated in glutamine supplemented medium and the following molecules have been injected subsequently: glucose (glc), oligomycin (olig.), and 2-deoxyglucose (2DG) (mean ± SD; *n* = 3, statistical analysis were performed by two-way ANOVA compared to A375-v with a 95% interval of confidence followed by Bonferroni’s post-test. *P < 0.05). **d** Immunoblotting of hexokinase 1 (HK1), hexokinase 2 (HK2), aldolase A (ALDO A), glyceraldehyde-3-phosphate dehydrogenase (GAPDH), and lactate dehydrogenase A (LDHA) expression in A375-v cells compared to A375RIV1. Actin served as loading control. **e** Colony-forming ability of A375-v and A375RIV1 exposed to mitochondrial pyruvate carrier (MPC) inhibitor UK5099 in full medium for 7 days. Pictures are representative of three independant experiments

In previous work, we and others have observed an increase of mitochondrial oxidative phosphorylation (OXPHOS) in melanoma cell lines resistant to BRAFi^9,10^. In agreement with our previous results, tumor biopsies from A375RIV1-engrafted mice also exhibited an increase in oxygen consumption compared to those from A375-v or A375-engrafted mice (Fig. 2a). Increase in OXPHOS activity was not associated with modification of mitochondrial content as judged by mtDNA quantification in resistant cells (Fig. 2b). Because the pyruvate dehydrogenase complex is a key modulator of mitochondrial activity through pyruvate oxidation in melanoma^14^, we studied its regulation by specific kinases, which results in inhibitory phosphorylation. No differences in the level of PDH phosphorylation at Ser293 and in PDK1/PDK3 expression were seen between A375-v and A375RIV1 (Fig. 2c). Moreover, the PDK inhibitor, DCA, increased OXPHOS to the same extent in both cell lines (Fig. 2c), suggesting that PDK/PDH axis per se cannot explain increased mitochondrial activity observed in resistant melanoma. Moreover, isolated mitochondria from A375RIV1 or A375-v tumors exhibited similar oxygen consumption rates regardless of the substrates (Fig. 2d). So we hypothesized that increase in OXPHOS found in melanoma cells resistant to BRAFi could be consecutive to glutamine oxidation as previously described by Marais and coll^10^. Indeed, the resistant cells, A375RIV1, consumed more glutamine than their sensitive counterpart, A375-v, and were more sensitive to glutamine withdrawal or glutamine inhibition by BPTES confirming glutamine addiction for proliferation of melanoma cells resistant to BRAFi (Fig. 2e–g). Together, these results confirmed that mitochondrial metabolism is drastically modified in BRAFi-resistant cells. We then examined glucose metabolism in resistant cells (Fig. 3). Prior drug treatment, there was no difference in glucose uptake examined in vivo by PET-Scan (Fig. 3a) between A375-v and A375RIV1 tumors. At the opposite, we have observed in vemurafenib-treated mice a decrease of glucose uptake in A375-v but not in A375RIV1 tumors confirming resistant phenotype. The same results have been also obtained in vitro, confirming that this metabolic behavior is maintained in cell lines (Fig. 3b).

In order to go further, we have measured glycolytic activity in real time by measuring the extracellular acidification rate using the XF24e Seahorse apparatus (Fig. 3c). Interestingly, we observed that basal glycolytic activity is significantly reduced in A375RIV1 cells compared to A375-v. However, after treatment with the ATP-synthase inhibitor, oligomycin A, both cell lines increased glycolytic activity at the level of that of A375rho0^5^, a cell line deficient in OXPHOS, which relies mainly on glycolysis suggesting that glycolytic pathway is not altered in A375RIV1-resistant cells. Moreover, no obvious differences in the expression of key glycolytic enzymes were observed between A375-v and A375RIV1 cells (Fig. 3d). These results suggest that pyruvate derived from glycolysis could be redirected in mitochondria in BRAFi-resistant melanoma cells. In agreement with this hypothesis, A375RIV1 cells were more prone than A375-v to growth inhibition induced by UK5099, a well-known inhibitor of mitochondrial pyruvate carrier (Fig. 3e). This result suggests that mitochondrial pyruvate metabolism could be important to support anabolism or survival pathway in A375RIV1 cells.

**Fig. 4:**
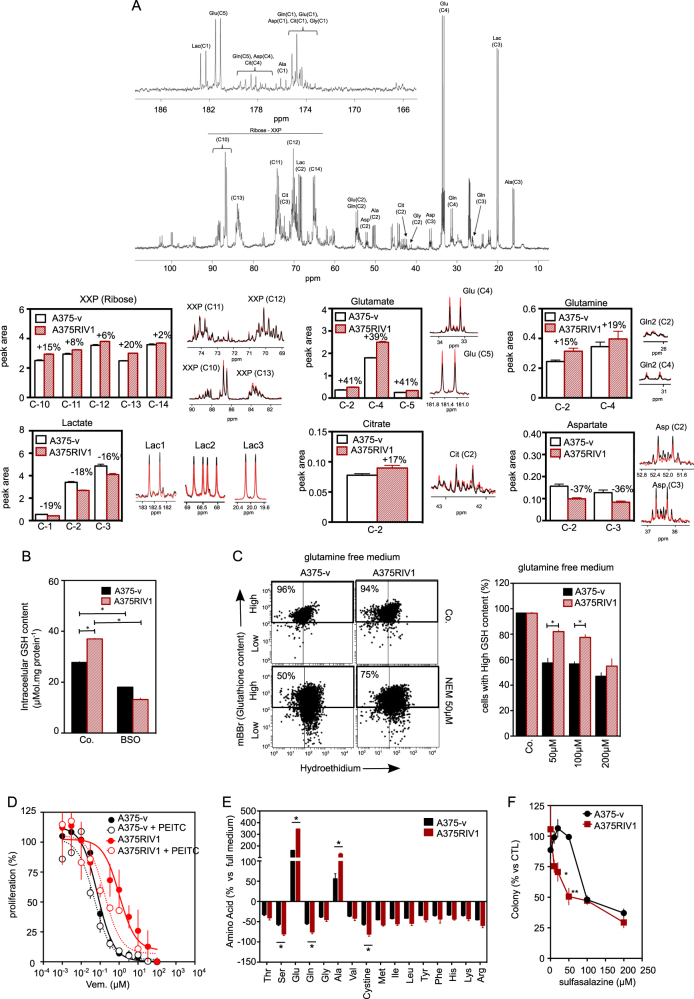
Mitochondrial metabolism favorizes de novo glutamate synthesis and related glutathione content. **a** Comparative steady-state isotopomer profiling of A375-v and A375RIV1 cells via ^13^C NMR analysis following 24 h incubation with [U-^13^C]-glucose. The percentage indicates the variation of the [U-^13^C_6_]-glucose-related metabolites of A375RIV1 compared to A375-v. **b** Determination of glutathione content by CPLH in A375-v and A375RIV1 as described in materials and methods (mean ± SD; *n* = 3, statistical analysis was performed by two-way ANOVA with a 95% interval of confidence followed by Bonferroni’s post-test. *P < 0.05). **c** Determination of oxidative stress with hydroethidium (HE) and glutathione content with monobromobimane (mBBR) in A375-v and A375RIV1. A375-v and A375RIV1 cells have grown for 24 h in glutamine-free medium (supplemented with glucose) and cells have been treated with *N*-ethyl maleimide (0, 50, 100, and 200 µM) for 24 h. Fluorescences of mBBR and HE have been assessed by flow cytometry. Results are representing as dot plot divided in two gates: cells with high GSH content are observed in upper gate; cells with low GSH content are observed in lower gate. Left panel: representative cytofluorimetric profile of experiment performed with A375-v and A375RIV1 treated with NEM 50 µM for 24 h. Right panel: histogram A375-v and A375RIV1 treated with increasing concentration of NEM (50, 100, and 200 µM) for 24 h (mean ± SD; *n* = 3, statistical analysis was performed by two-way ANOVA with a 95% interval of confidence followed by Bonferroni’s post-test. *P < 0.05). **d** Proliferation of A375-v (black line) or A375RIV1 (red line) cells treated with increasing concentration of vemurafenib for 72 h. When indicated, cell lines have been conjointly treated with PEITC (5 µM). Cell number has been determined using Cyquant reactif and measured with a spectrofluorimeter. **e** A375-v or A375RIV1 have grown for 24 h in full medium and amino acid concentration has been compared to a fresh full medium (mean ± SD; *n* = 3, Student’s *t* test). **f** Colony-forming ability of A375-v and A375RIV1 grown in full medium and treated with sulfasalazine as indicated for 7 days. The values represent the mean ± SD of three independent experiments. Statistical analysis was performed by two-way ANOVA with a 95% interval of confidence followed by Bonferroni’s post-test. *P < 0.05 and **P < 0.01)

**Fig. 5:**
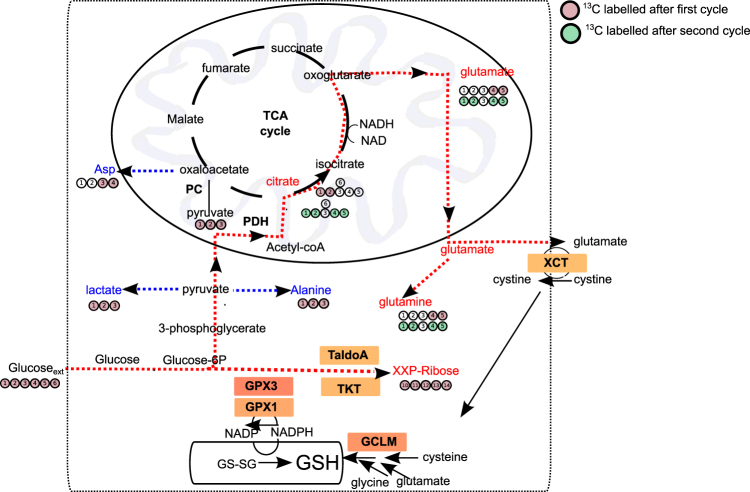
Biochemical pathway in A375RIV1 in favor of GSH synthesis. Gene overexpressed in A375RIV1 compared to A375-v are visualized in boxes (see Fig. 6a for results). Glucose-related metabolites that have been measured with higher concentration in A375RIV1 compared to A375-v are indicated in red and measured with lower concentrations are indicated in blue (see Fig. 4a, e for results)

To get further insights into the metabolism of glucose-derived pyruvate, we quantified metabolites by NMR in A375-v and A375RIV1 growing in DMEM medium supplemented with U-^13^C-glucose (Figs. 4a and 5). ^13^C enrichment of nucleotides (XXP) from glucose was significantly higher in A375RIV1 than in A375-v, suggesting a more active pentose phosphate pathway in resistant cells. Conversely, ^13^C incorporation into lactate was decreased in A375RIV1 in agreement with results of Fig. 3c. A375RIV1 exhibited higher ^13^C accumulation of citrate, glutamate, and glutamine indicating an increase of glucose-derived pyruvate to feed TCA cycle. Besides, aspartic acid synthesized from oxaloacetate-derived TCA is decreased suggesting a downregulation of this pathway in favor of glutamate synthesis. Together, these results reveal unexpected metabolic complexity of resistant cells with glutamate synthetized de novo from glucose as prominent characteristic of melanoma resistant to BRAFi. Glutamate is directly utilized in the biosynthesis of glutathione, the major defense against oxidative stress in melanoma. As expected, GSH content was higher in A375RIV1 than in A375-v (Fig. 4b). As positive control, A375-v or A375RIV1 were treated with DL-buthionine-[S,R]-sulfoximine (BSO), a known GSH-depleting agent. To test if glucose-derived glutamate is involved in GSH synthesis, A375-v and A375RIV1 cells were grown in glutamine-free medium supplemented with glucose then GSH content was determined by flow cytometry after progressive depletion in GSH content induced by increasing concentration of *N*-ethyl-maleimide (NEM) (Fig. 4c). Irrespective of NEM concentrations, A375RIV1 maintained higher GSH content than A375-v in glutamine-free glucose medium, suggesting a predominant role of glucose-derived glutamate in GSH synthesis in BRAFi-resistant cells. GSH content seems to be important for A375RIV1 survival cells because PEITC^15^, an inhibitor of glutathione S-transferase, decreases vemurafenib-acquired resistance of A375RIV1 (Fig. 4d). Another way in which glutamate participates to glutathione biosynthesis is indirectly through facilitating the uptake of cystine via the xCT transporter, which is coupled to the efflux of glutamate. Using ^12^C-glucose, we have measured AA concentration in the medium (Fig. 4e). In agreement with Fig. 2e, we found a more important consumption of glutamine in the medium of A375RIV1 compared to A375-v. We have also measured an important increase of glutamate release in the medium of A375RIV1 and important consumption of cystine (Fig. 4e). This metabolic behavior seems to be important for A375RIV1 cells because sulfasalazine, an inhibitor of X_C_^−^ glutamate/cystine exchange, inhibited preferentially the growth of A375RIV1 compared to A375-v (Fig. 4f). Together these results indicate that resistant cells use glucose-derived glutamate to support glutathione synthesis through several complementary pathways.

**Fig. 6:**
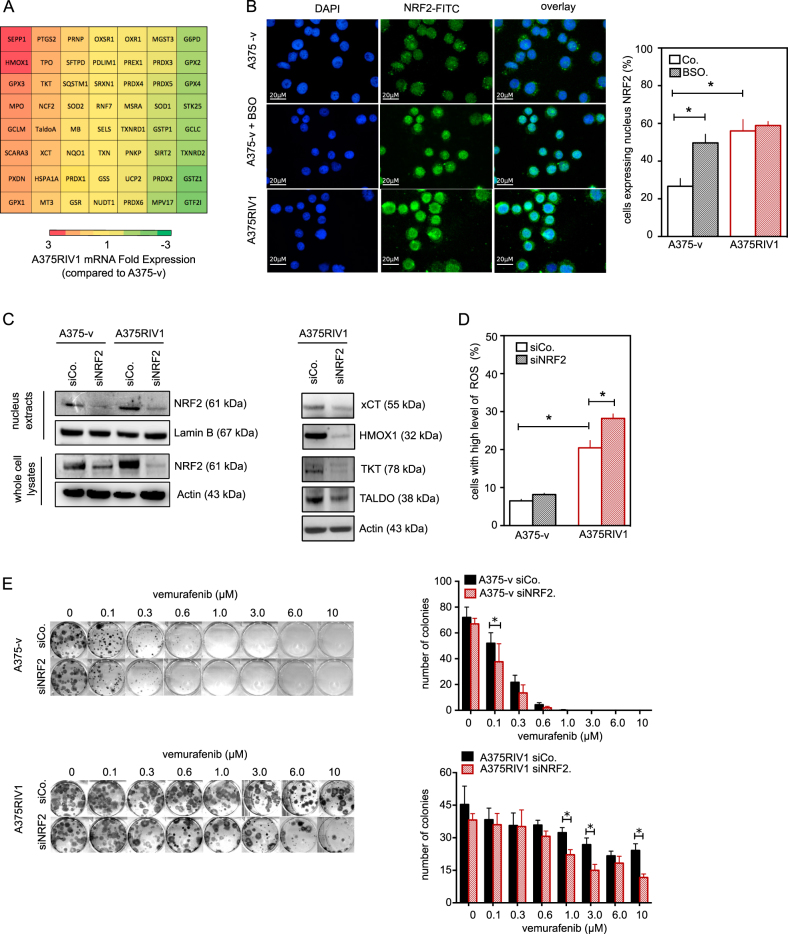
NRF2 promotes antioxydant response in MAPK-resistant melanoma. **a** mRNA expressions of genes implicated in oxidative stress status have been assessed by multiplex PCR in A375RIV1 compared to A375-v. **b** Immunofluorescence of NRF2 in A375-v and A375RIV1 cells. BSO has been used as positive control in A375-v to favorize NRF2 nuclear accumulation. DAPI staining has been used to visualize nucleus compartment. Left panel: representative pictures of three independent experiments. Right panel: percentage of A375-v or A375RIV1 treated or not with BSO exhibiting nuclear NRF2 labeling in nucleus. The values represent the mean ± SD of three independent experiments. Statistical analysis was performed by two-way ANOVA with a 95% interval of confidence followed by Bonferroni’s post-test. *P < 0.05). **c** Left panel: immunoblotting of NRF2 in isolated nuclei or in whole-cells lysates of non-specific scramble control (SiCo.) or NRF2 silencing (SiNRF2) cells. Actin or Lamin B served as loading control. Right panel: immunoblotting of anionic amino acid transporter light chain, xCT, or Slc7a11 (the specific subunit of system X_C_^−^), Heme oxygenase 1 (HMOX1), transketolase (TKT), and transaldolase (TALDO) in si-control or si-NRF2 treated-A375RIV1 cells. Actin served as loading control. **d** ROS production in A375-v or A375RIV1 has been evaluated by flow cytometry after hydroethidium (HE) labeling in si-control or siNRF2-treated cells. **e** Left panel: colony-forming ability of A375-v and A375RIV1 grown in full medium and treated with vemurafenib as indicated for 10 days. Prior drug treatment, cells have been transfected with non-specific scramble control (SiCo.) or with SiRNA NRF2 (SiNRF2) as indicated. Representative pictures of three independent experiments. Right panel: the number of colonies has been counted for each condition and was indicated in the histogram. The values represent the mean ± SD of three independent experiments. Statistical analysis was performed by two-way ANOVA with a 95% interval of confidence followed by Bonferroni’s post-test. *P < 0.05

Also, consistent with this model is the observation that A375RIV1 overexpressed many antioxidant genes including those involved in GSH synthesis as xCT and GCLM, in GSH regeneration as glutathione peroxidase GPX1 and GPX2 and in NAPDH production (Transaldolase A and Transketolase) (Fig. 6a). Since the nuclear factor erythroid 2-related factor 2 (NRF2) is a transcription factor that regulates the expression of antioxidant response genes including those upregulated in A375RIV1 (Fig. 6a), we have first determined the nuclear levels of NRF2 (i.e., the active form) in both cell lines by immunofluorescence and western blot (Fig. 6b, c). As a positive control, A375-v or A375RIV1 were treated with BSO, known to induce NRF2 activation^16^. A375RIV1 exhibited higher expression levels of NRF2 in the total cell lysates as well as in the nuclear fraction than A375-v. In addition, the knockdown of NRF2 by siRNA significantly decreased the protein expression of the NRF2 targets: xCT, XMOX1, TKT, and TALDO (Fig. 6c) as well as it increased ROS production in A375RIV1 (Fig. 6d). These results demonstrate that NRF2 contributes to support the antioxidant defense in BRAFi-resistant melanoma. Interestingly, NRF2 silencing partially reversed vemurafenib resistance of A375RIV1 cells as judged by clonogenic assay in Fig. 6e. In conclusion, all these results demonstrated that glucose-derived glutamate contributes to increase the antioxidant defense in BRAFi-resistant melanoma cells in an NRF2-dependent manner, contributing to the survival to anti-BRAF therapy.

## Discussion

BRAFi-resistant melanoma cells are characterized by an increase in mitochondrial oxidative phosphorylation, which enhances the level of oxidative stress and renders them more sensitive to cell death induced by pro-oxidative drugs^5^. Here, we demonstrate that melanoma cells resistant to MAPK inhibitors rewire glucose metabolism and activate integrated NRF2-dependent antioxidant responses for protection against oxidative stress (Fig. 5).

First, we established in vivo preclinical murine models that accurately recapitulate the acquisition of resistance to the BRAFi, vemurafenib. To model the emergence of vemurafenib resistance, we generated BRAF-mutated A375 cells resistant to vemurafenib (A375RIV1) using an in vivo long-term vemurafenib-treated-xenograft model (Fig. 1). As observed in patients^17^, mice-derived A375RIV1 have also acquired cross resistance to numerous MAPK inhibitors including the compounds dabrafenib, trametinib, cobimetinib alone, and in combination. Tumor biopsies confirmed that the acquisition of resistance is associated with an increase in mitochondrial oxidative phosphorylation as well as the importance of glutamine metabolism in these cells. This observation supports the previous studies performed with resistant melanoma cell lines obtained in vitro with increasing drug concentration^9,10^.

We found that glucose-derived pyruvate is not mostly converted into lactate in BRAFi-resistant melanoma but feeds preferentially mitochondrial TCA cycle. In line with our results, Delgano-Goni et al. have shown recently that vemurafenib-treated WM266.4 cells also reduced de novo synthesis of lactate in favor of mitochondrial pyruvate uptake through pyruvate carboxylase. Surprisingly, we also observed the significant contribution of glucose to glutamate synthesis in resistant cells. This metabolic behavior has been already observed in glioblastoma in vivo^18,19^ where glucose-derived glutamate is used for de novo synthesis of glutamine. In melanoma resistant to BRAFi, we showed that a part of the glutamate produced from TCA cycle-derived carbons was released in the extracellular environment. To explain the high level of glutamate release, we found that resistant melanoma cells overexpressed xCT (*a.k.a*. SLC7a11), a component of cysteine/glutamate exchange transport (X_C_^−^ system) mediating cysteine entry into cell in exchange for glutamate. The activity of system X_C_^−^ contributes to the maintenance of intracellular GSH levels^20^. Thus, the overexpression of xCT observed in BRAFi-resistant melanoma is in agreement with their higher GSH content.

Because xCT expression is controlled by the transcription factor NRF2 in various cancer cell lines^21^, we decided to measure expression levels of NRF2. In physiological conditions, NRF2 binds to its repressor protein Keap1 (Kelch ECH associating protein) and is subjected to continuous ubiquitination and proteosomal degradation. Under oxidative stress, conformation of Keap1 is modified leading to NRF2 release, which translocates to the nucleus then after heterodimerization with the Maf proteins bind to the antioxidant response element (ARE) of target genes. We demonstrated that A375 melanoma cells exhibited a significant level of nuclear NRF2 accumulation. This observation is in agreement with the fact that BRAF oncogenes activate the NRF2 antioxidant program^22^. Interestingly, NRF2 accumulation was even higher in BRAFi-resistant melanoma cells (Fig. 6b, c). The metabolic phenotype of BRAFi-resistant melanoma cells could be strongly influenced by NRF2 accumulation. Indeed, transcriptomic analysis has identified many NRF2 target genes, which are upregulated in BRAFi-resistant cells including genes implicated in iron sequestration (HMOX1), in the pentose phosphate pathway, and NADPH production (TKT and TALDO) as well as in GSH production and regeneration (GCLM and xCT). NRF-2 accumulation could be also implicate in increase of exogenous glutamine consumption as shown by Sayin et al.^23^. Besides, NRF2 could also maintain mitochondrial metabolism in BRAFi-resistant melanoma cells for several reasons. First, it has been shown that the knockdown of NRF2 decreases basal mitochondrial respiration and spare respiratory capacity in numerous colon cancer cell lines^24^. Second, NRF2 controls substrate availability for mitochondrial respiration^25^. NRF2 affects the efficiency of mitochondrial fatty acid oxidation in mouse embryonic fibroblasts (MEF)^26^. Mitochondrial oxidation of fatty acids (palmitic or hexanoic) is depressed in NRF2 knockout MEF and accelerated when NRF2 is constitutively active.

Finally, we decided to evaluate the role of glutathione metabolism in the BRAFi resistance context. First, we have used PEITC, a compound of the members of the isothiocyanate family. Its conjugation with glutathione is catalyzed by glutathione S-transferase, leading to a rapid depletion of glutathione^15^. We have shown that PEITC is able to resensitize to vemurafenib BRAFi-resistant melanoma cell lines confirming the important role of glutathione in their survival. But PEITC is also able to induce disruption of the mitochondrial electron transport complex I and to inhibit mitochondrial respiration in some cancer cells lines as leukemia cells^27^, thereby by this mechanism it could also increase cytotoxicity of BRAFi as seen with other complex I inhibitors metformin or phenformin^11,28^. To inhibit GSH metabolism, we have also used sulfasalazine, an xCT inhibitor; we found that this drug delay in vitro the growth of BRAFi-resistant melanoma. This FDA-approved drug is used in the treatment of inflammatory bowel disease, but preclinical study has also shown that this drug increases also chemosensitivity to multiple drugs^29^. Erastin could be also another potential drug to increase BRAFi therapy, due to the fact that it is an X_C_^−^ inhibitor and this compound exhibits greater lethality in cancer cells harboring mutations in HRAS, KRAS, or BRAF^30,31^.

In conclusion, we found that BRAFi-resistant melanoma adapt their mitochondrial metabolism to favorize glucose-derived glutamate synthesis, cysteine uptake trough xCT, and glutathione synthesis implicating NRF2 pathway. Glutathione level substaines survival of these cells in this oxidative stress condition. Drug targeting glutathione metabolism could be useful to delay the emergence of resistance to anti-MAPK therapy.

## Materials and methods

### Chemicals

All chemicals were purchased from Sigma-Aldrich (St Louis, MO, USA). Vemurafenib, cobimetinib, trametinib, and dabrafenib were from SelleckChem (Euromedex, Souffelweyersheim, France)

### Cell lines

A375 human melanoma cell lines were purchased from the American Type Culture Collection. The original cells were short tandem repeat DNA profiled (IGNA, Nantes, France), were grown in bulk, and were never passaged for more than 6 weeks. A375 cell lines have been found to harbor BRAFV600E mutation. A375, A375-v, A375RIV1, and A375RIV2 were maintained in RPMI with 10% FCS. All cell lines were periodically tested for mycoplasma contamination.

Cells were transfected with siRNA targeting NRF2 (sc-37030, Santa Cruz Biotechnology, Santa Cruz, CA, USA) or a non-targeting control siRNA (sc-37707). Transfections were performed in Opti-MEM (Invitrogen) using Lipofectamine plus (Invitrogen) following the manufacturer’s instructions.

### In vivo study

All procedures with animals were conducted according to the Institutional guidelines. Severe combined immunodeficient female mice (SCID), 6- to 8-week-old, under isoflurane anesthesia were injected with 2 × 10^6^ A375 cells, mixed (1:1 volume) with BD Matrigel Basement Membrane Matrix. When tumors reached 100–250 mm^3^, the two groups were divided randomly (*n* = 25) and mice were treated with saline solution or with vermurafenib administrated by oral gavage (75 mg/kg/d). [^18^F]FDG PET scans have been realized as described previously^32^.

### Histology

Tumor specimens were stained with H&E. For in situ determination of cell proliferation, sections were analyzed with an antibody to Ki67. Stainings with antibodies against the highly characterized melanoma markers S100B have been used to confirm cellular pattern of melanoma^33^.

### Clonogenic assay and proliferation

Clonogenic assay was realized with cells (500/well) seeded into 6-well plates and treated with indicated doses of drug in culture medium. After 7 days of culture, colonies were stained with crystal violet, digital images were taken, then colonies were de-stained in acetic acid (30%) before densitometric quantification with the SAFAS UVMc2 spectrophotometer (Safas Monaco).

Cell proliferation was determined by cell numbers recorded after being seeded, using CyQuant direct proliferation kit from Invitrogen (Carlsbad, CA, USA).

### Glucose and glutamine measurements

Glucose was measured in the extracellular medium using a SYNCHRON LX20 Clinical system (Beckman Coulter, Fullerton, CA USA). Glutamine was measured using glutamine colorimetric assay kit (BioVision) following the manufacturer’s instructions.

### Immunoblotting

Cell lysates were prepared as described previously^34^ then 20 µg proteins were separated on a 4–12% SDS-PAGE then transferred to nitrocellulose membrane. After blocking for 1 h in 10% milk in TBS Tween buffer, membranes were probed with antibodies as described in Supplementary Table 1. Horseradish peroxidase-conjugated secondary antibodies from Rockland Immunochemicals Inc. (Limerick, PA) were used at 1:2000 for 1 h then detection was carried out by enhanced chemoluminescence.

### Assessment of oxygen consumption and glycolysis activity

Respiratory capacity and glycolytic activity of cells were performed with the Seahorse XF24e Extracellular Flux Analyser (Seahorse Bioscience, Billerica, MA, USA) on attached cells as described^9^. Briefly, 2 × 10^4^ cells/well were seeded in XF24 V7 microplates for 24 h before drug treatment. To determine oxygen consumption rate (OCR), cells were resuspended in DMEM (D5030, Sigma-Aldrich) with l-glutamine (2 mM) and d-glucose (10 mM) and the following drugs were added: 1 µM oligomycin, 0.25–0.5 µM FCCP, 1 µM rotenone, and 1 µM antimycin A. To determine extracellular acidification rate (ECAR), cells were resuspended in glucose-free DMEM (D5030, Sigma-Aldrich) with l-glutamine (2 mM) and the following compounds were added: d-glucose (10 mM), oligomycin (1 µM), and 2DG (10 mM).

### ^13^C NMR isotopomer profiling after treatment of cells with [U-^13^C]-glucose

A375-v and A375RIV1 cells were incubated for 24 h in DMEM medium containing [U-^13^C]-glucose (10 mM). Metabolites were extracted^35^ and redissolved in 550 µl of phosphate buffer (0.2 M; D_2_O) at pH 7.4. 1D ^13^C NMR experiments were acquired with power-gated proton decoupling (3.1 kHz, Waltz-16) at 298 K on a Bruker Avance 500 equipped with a 5-mm cryoprobe. Acquisition parameters were as follow: relaxation delay 2 s, acquisition time 0.93 s, spectral width 35.2 kHz (280 ppm), and number of scans 20 K. ^13^C NMR spectra were processed using the Bruker TopSpin software 3.1, integrated with the KnowItAll® software (BIO-RAD) and normalized using the external standard and the amount of proteins in the sample as previously described^35^.

### Amino acid measurements

Amino acids concentration assay (μmol/l) was performed by high-performance liquid chromatography (Shimadzu C18 column, Kyoto, Japan)—tandem mass spectrometry (AB Sciex 3200 Qtrap, Framingham, MA) using the aTRAQ kit for amino acid analysis of physiological fluids (AB Sciex). Acquisition in the mass spectrometer was achieved by multiple reaction monitoring. Data recording and analysis were performed with Analyst software, v.1.6 (AB Sciex).

### Glutathione status

Glutathione status (i.e., glutathione disulfide, GSSG/reduced glutathione, GSH) was determined in cell pellets by using high-performance liquid chromatography with fluorescence detection, as published^36^.

### Cytofluorometric analysis

Evaluation of cell viability was performed with propidium iodide staining (5 μg/ml, 15 min, 4 °C) (P4864, Sigma-Aldrich, St. Louis, MO, USA). Changes in glutathione content were analyzed with the monobromobimane (100 nM, 30 min, 37 °C) (M1378, ThermoFisher Scientific, Grand Island, NY). Detection of ROS was determined using hydroethidium probe (2.5 µM, 30 min, 37 °C) (D1168, ThermoFisher Scientific, Grand Island, NY). Fluorescences were analyzed on a FACS LSR cytofluorometer (Beckton Dickinson).

### PCR analysis

Quantitative detection of mRNA was performed by real-time PCR with RT² Profiler™ PCR Array Human Oxidative Stress (PAHS-065Z) using the Lightcycler 480 detector (Roche Applied Science, Manheim Germany). Data analysis is based on QIAGEN Web software using the ΔΔCT method with normalization of the raw data to either housekeeping genes.

Mitochondrial DNA (mtDNA) relative copies number was assessed by measuring RNA level relative of mtDNA-encoded ND2 gene expression or ATPase6 gene expression. All samples have been normalized by nuclear DNA-encoded ATPsynthB RNA (reference genes). The comparative CT method was used for relative quantification of gene expression. Differences in the CT values (dCT) of the transcript of interest and reference genes were used to determine the relative expression of the gene in each sample^37^.

### Microscopic imaging

For confocal microscopy, cells were grown on coverslips and then stained with anti-NRF2 (NRF2 C-20 SC722, Santa Cruz Biotechnology, Santa Cruz, CA, USA). Immediately before examination, cells were counterstained with DAPI (D1306, ThermoFisher Scientific, Grand Island, NY). Before experiments, cells were seeded on 24 mm glass coverslip for 24 h before microscopic analysis (Leica DMR, Heidelberg, Germany). The confocal microscope was equipped with a 488 nm Argon laser, 561-nm diode-pumped solid-state laser and a 405-nm ultraviolet laser. Images were acquired using an oil ×40 Plan-NEOFLUAR objective (1.3 NA) and an oil ×63 Plan-APOCHROMAT objective (1.4 NA).

### Statistical analysis

Statistics were performed with GraphPad Prism® version 6.0 (GraphPad Software, San Diego, CA, USA). Data are presented as the mean ± SD. Differences between measurements in groups were determined by Student's *t* test, a one-way ANOVA or two-way ANOVA with Bonferroni post-test analysis as indicated. *P* ≤ 0.05 was considered significant with *P* ≤ 0.05 indicated with (*), *P* ≤ 0.01 with (**), *P* ≤ 0.001 with (***).

## Electronic supplementary material

Supplemental material
