# Supplementary material for: Glucose metabolism and NRF2 coordinate the antioxidant response in melanoma resistant to MAPK inhibitors
Source: Cell Death Dis. 2018 Feb 27;9(3):325. doi: 10.1038/s41419-018-0340-4 (PMC5832419; doi:10.1038/s41419-018-0340-4)
Supplement: Supplementary file 1 — Supplemental material [file 41419_2018_340_MOESM1_ESM.pdf]

Supplemental material: list of antibodies

HXK1, Hexokinase I, sc-46695, Santa Cruz Biotechnologies INC; HK2, Hexokinase II (C64G5), #2867, Cell Signaling technology; Aldo, Aldolase, #3188, Cell Signaling technology; GAPDH (FL-335), Glyceraldehyde 3 phosphate dehydrogenase, sc-25778, Santa Cruz Biotechnologies Inc; LDHA, Lactate dehydrogenase A (E-9), sc-137243, Santa Cruz Biotechnologies INC; B-Actin (C4), sc-47778, Santa Cruz Biotechnologies INC; NRF2 (C-20), NF-E2 DNA binding protein, sc-722, Santa Cruz Biotechnologies INC; xCT, ab8417, Abcam, H; Heme Oxygenase 1 (A-3), sc-136960, Santa Cruz Biotechnologies INC; Transketolase (K-20), sc-46552, Santa Cruz Biotechnologies INC, Transaldolase, sc-51439, Santa Cruz Biotechnologies Inc; PDK1 (2H3AA11), Mitochondrial pyruvate dehydrogenase kinase 1, ab110335, Abcam; PDK3 (RR-2), sc-100535, Santa Cruz Biotechnologies INC; pSer293-PDH, Pyruvate Dehydrogenase E1-alpha subunit (phospho S293) antibody (ab92696), Abcam ; PDHE1A, Pyruvate Dehydrogenase E1-alpha subunit antibody [EPR11098] (ab168379)
